# Role of surface oxygen-containing functional groups of graphene oxide quantum dots on amyloid fibrillation of two model proteins

**DOI:** 10.1371/journal.pone.0244296

**Published:** 2020-12-23

**Authors:** Ebrahim Rostampour Ghareghozloo, Mohsen Mahdavimehr, Ali Akbar Meratan, Nasser Nikfarjam, Atiyeh Ghasemi, Bentolhoda Katebi, Mohsen Nemat-Gorgani

**Affiliations:** 1 Department of Biological Sciences, Institute for Advanced Studies in Basic Sciences (IASBS), Zanjan, Iran; 2 Department of Chemistry, Institute for Advanced Studies in Basic Sciences (IASBS), Zanjan, Iran; 3 Institute of Biochemistry and Biophysics, University of Tehran, Tehran, Iran; 4 Stanford Genome Technology Center, Stanford University, Palo Alto, CA, United States of America; Rijksuniversiteit Groningen, NETHERLANDS

## Abstract

There are many reports demonstrating that various derivatives of carbon nanoparticles are effective inhibitors of protein aggregation. As surface structural features of nanoparticles play a key role on modulating amyloid fibrillation process, in the present *in vitro* study, bovine insulin and hen egg white lysozyme (HEWL) were selected as two model proteins to investigate the reducing effect of graphene oxide quantum dots (GOQDs) on their assembly under amyloidogenic conditions. GOQDs were prepared through direct pyrolysis of citric acid, and the reduction step was carried out using ascorbic acid. The prepared nanoparticles were characterized by UV-Vis, X-ray photoelectron, and FT-IR spectroscopies, transmission electron and atomic force microscopies, zeta potential measurement, and Nile red fluorescence assay. They showed the tendencies to modulate the assembly of the proteins through different mechanisms. While GOQDs appeared to have the capacity to inhibit fibrillation, the presence of reduced GOQDs (rGOQDs) was found to promote protein assembly via shortening the nucleation phase, as suggested by ThT fluorescence data. Moreover, the structures produced in the presence of GOQDs or rGOQDs were totally nontoxic. We suggest that surface properties of these particles may be part of the differences in their mechanism(s) of action.

## Introduction

**Fig 1:**
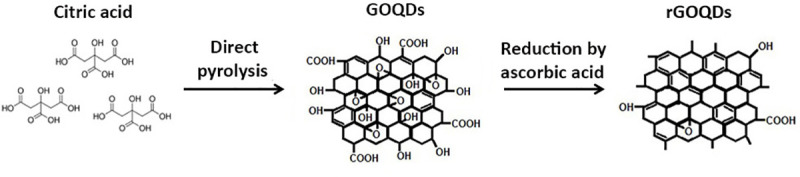
Schematic presentation of GOQDs synthesis and reduction.

Misfolding and aggregation of proteins into fibrillar structures is associated with a large number of disorders including Alzheimer's and Parkinson's diseases and type 2 diabetes [1]. All these conditions are characterized, in part, by the presence of long-unbranched amyloid fibrils with a cross-β structure, indicating a common mechanism of aggregation [2]. However, amyloid fibril formation is a complicated process and appears to involve abnormal assembly of proteins from their soluble monomers into small oligomers, protofibrils, and eventually insoluble mature fibrils [3, 4]. Thus small oligomers and protofibrillar species, produced during the aggregation process, are considered as a potential therapeutic target for treatment of amyloid-related diseases. Common approaches explored for inhibition of protein aggregation can be divided into four major classes including: (I) naturally occurring small molecules from different sources, including vegetables and fruits [5], (II) proteins, peptides, and peptide analogues [6–8], (III) antibodies [9], and (IV) series of nanoparticles [10], including polymeric nanoparticles [11], functionalized nanoparticles [12, 13] and carbon nanoparticles [14–18]. Recently, carbon nanoparticles have received considerable attention due to their ability to cross biological membranes [19, 20] and even targeting amyloid plaques via crossing the blood-brain barrier [21–25]. Among them, graphene oxide (GO) derivatives have attracted a lot of attention due to their non-toxicity, flexible molecular structure, easy functionalization, excellent biocompatibility, and low cost of preparation [26]. Additionally, they have the ability to form strong non-covalent interactions (including π-π stacking, electrostatic forces, and hydrogen bonding) with adsorbed biomolecules and easily form stable dispersions in water and organic solvents [26–28]. Graphene oxide quantum dots (GOQDs) are atomically a thin sheet of GOs with sizes frequently around 10 nm. Like GOs, GOQDs are covalently decorated with oxygen-containing functional groups at their edges and basal plane [29]. These features provide these materials with applicability in versatile fields such as drug delivery, cellular imaging, and tissue engineering. Generally, two main methods have been developed for GOQDs preparation, known as “top-down” and “bottom-up” [29]. Most of the top-down methods require special equipment and critical conditions for their synthesis in good yield and desired size. These methods include chemical oxidation treatment of carbon fibers and carbon black, carving graphite crystallites by high resolution electron beam lithography, cage opening of the fullerene on ruthenium surfaces, and cutting GO through hydrothermal, re-oxidation or electrochemical routes. While in the bottom-up methods, some organic precursors are carbonized by thermal treatment, which usually leads to precise size control and uniform morphology [29]. Some reports have suggested that the surface of nanomaterials, including GO derivatives, become covered by biomolecules such as proteins upon their entrance into a biological matrix [30, 31]. Accordingly, studies on the inhibition of amyloid fibrillation of various peptides and proteins by GO derivatives have been the focus of much attention [17, 20, 32–34]. Clearly, both surface hydrophobicity and functional groups on the surface of GO, may participate in non-covalent interactions with proteins [17], and consequently play a key role on their anti-amyloidogenic properties. In the present study, GOQDs were prepared through a simple and facile bottom-up strategy, by using citric acid (CA) as a common organic precursor, followed by reduction with ascorbic acid (AA) (Fig 1).

Then, reduction effect of oxygen-containing functional groups of GOQDs on the amyloid fibrillation of two well-known model proteins, including bovine insulin and hen egg white lysozyme (HEWL), was investigated. Both bovine insulin and HEWL are well-characterized proteins that under *in vitro* conditions form amyloid fibrils [35, 36], and are routinely used for studies related to amyloid fibrillation inhibition. Moreover, the structural similarity of these proteins with human insulin and human lysozyme, which are associated with the injection-localized amyloidosis, and hereditary non-neuropathic systemic amyloidosis, respectively [37–39], make them ideal model structures for amyloid-related studies. We employed UV-Vis, X-ray photoelectron, and fourier transform infrared (FT-IR) spectroscopies, transmission electron microscopy (TEM), atomic force microscopy (AFM), zeta potential measurement, and Nile red fluorescence assay to characterize structural and morphological features of GOQDs and reduced GOQDs (rGOQDs). This was followed by using a range of amyloid-specific techniques, including Thioflavin T (ThT) and ANS fluorescence assays, Congo red (CR) binding measurement, circular dichroism (CD) analysis, and AFM imaging to explore the capacity of these nanoparticles in modulating amyloid fibrillation of bovine insulin and HEWL. Our results clearly indicate the potency of GOQDs in inhibiting the fibrillation process and related cytotoxicity, in a dose-dependent manner. For samples incubated with rGOQDs, however, promotion of the assembly process was observed. We suggest that the anti-amyloidogenic mechanisms may be attributed to the differences in surface properties of the nanoparticles, as well as the structural characteristics of the proteins.

## Materials and methods

### Reagents

HEWL, bovine insulin, Thioflavin T (ThT), Congo red (CR), 1-anilino-naphthalene 8-sulfonate (ANS), Nile red, and 3-(4,5-dimethyl tiazol-2-yl)-2,5 diphenyl tetrazolium bromide (MTT), were purchased from Sigma (St. Louis, MO, USA). SH-SY5Y cells were a gift from Dr. Karima (Shahid Beheshti University of Medical Sciences, Tehran, Iran). The cell culture medium (DMEM-F12), fetal bovine serum (FBS), and penicillin–streptomycin antibiotics were purchased from Gibco BRL (Life Technology, Paisley, Scotland). Citric acid (CA), ascorbic acid (AA), and all other chemicals were obtained from Merck (Darmstadt, Germany) and were reagent grade.

### Synthesis and reduction of graphene oxide quantum dots

Graphene oxide quantum dots (GOQDs) were prepared as previously reported by Dong et al. using direct pyrolysis of CA [29]. Briefly, 5 g CA was added into a 100 ml round-bottom flask, and heated to 200 ^○^C for 15 min. A change in color from colorless to dark orange indicated formation of GOQDs. The solution was then neutralized by adding 200 ml of NaOH solution (250 mM), resulting in an aqueous solution of GOQDs, with a pH around 11. The obtained suspension was dialyzed (using cut-off 3 KDa dialysis tube) against distilled water for 24 h, and the resultant solution, containing the purified small GOQDs, was dried in an oven at 75 ^○^C. The obtained powder was grinded finely and stored at room temperature (RT) for subsequent experiments. For reduction of GOQDs, 100 mg of AA, known as a green reduction agent, was added to a 250 ml of GOQDs aqueous solution (0.4 mg/ml) adjusted at pH 11 under constant stirring, and the mixture was heated at 95 ^○^C for 3 days, with mild stirring [40, 41]. Finally, the colloidal suspension was centrifuged at 16000 rpm for 10 min to remove the unreacted AA and side-products. Further purification was achieved by repeating this process, using deionized water (DW). The purified rGOQDs were dried in an oven at 75°C, grinded finely and stored at RT for next experiments.

### Characterization of GOQDs and rGOQDs

Prepared GOQDs and rGOQDs were characterized by using a range of techniques. The reduction progress was confirmed by UV-Vis, X-ray photoelectron, and fourier-transform infrared (FT-IR) spectroscopies. UV-Vis absorption of aqueous solutions of GOQDs and rGOQDs (0.3 mg/ml) were acquired using Shimadzu UV-2550 spectrometer. The X-ray photoelectron spectroscopy (XPS) analysis for the GOQDs and rGOQDs was performed using a K-Alpha spectrometer. The FT-IR spectrum of samples was recorded on a EQUINOX 55 Bruker using preparing their KBr pellets from 450 to 4000 cm^-1^. TEM images were acquired by placing the aqueous suspension (~0.02 mg/ml) of GOQDs or rGOQDs on the carbon-coated copper grids, and blotted after 30 s. For AFM analysis, GOQDs or rGOQDs powders were diluted with DW to a final concentration of 2 mg/ml, and 10 μl of the diluted samples were placed on freshly cleaved mica, and dried at RT. Images were acquired using a quantitative AFM (ARA-AFM, Ara-Research Company, Iran) in a non-contact mode, and were analyzed using SPIP-6.7.5 software. The surface zeta potential of GOQDs and rGOQDs at a range of pH (1.5–8.5) was measured using a zeta potential analyzer (Brookhaven Instrument, Holtsville, NY 11742–1896, USA). Finally, changes in surface hydrophobicity of GOQDs upon chemical reduction were monitored by Nile red fluorescence measurement. Briefly, aliquots of GOQDs or rGOQDs solutions were diluted to a final concentration of 2 mg/ml in H_2_O containing 50 μM Nile red. Samples were excited at 530 nm and emission spectra were recorded from 540 to 800 nm, with 5 and 10 nm slit widths for excitation and emission, respectively.

### Amyloid fibril formation

Amyloid fibrillation of bovine insulin was induced by incubation of protein solution (prepared in H_2_O/HCl solution (pH 1.5) at a final concentration of 100 μM) at 57 ^○^C for 6 h, while being stirred at 500 rpm. HEWL amyloid fibrils were prepared as previously reported with slight modifications [42]. Briefly, the protein was dissolved in 50 mM glycine buffer (pH 2.2) to a final concentrations of 50 μM, and aliquots were incubated at 57 ^○^C while being stirred at 300 rpm to induce amyloid fibril formation. Stock solutions of GOQDs were prepared at 50 mg/ml, using DW as solvent, and were stored at RT until use; where they were stable for several months. However, due to high propensity of rGOQDs for precipitation, we used freshly prepared solutions of rGOQDs for our experiments.

### Amyloid fibril detection and characterization

For monitoring the growth of amyloid fibrils, ThT fluorescence intensity of protein samples incubated without or with various concentrations of GOQDs or rGOQDs was determined using a mixture of 2 μM protein solutions and 10 μM ThT, with fixed excitation at 440 nm and emission at 485 nm. The acquired data from ThT fluorescence measurements were fitted to the sigmoid curve and kinetics parameters were determined according the equation described by Nielsen et al. [43]. For ANS fluorescence measurements, aliquots of protein solutions were removed at different time intervals, and diluted to a final concentration of 2 μM, using H_2_O containing 20 μM ANS. Samples were excited at 350 nm and emission spectra were recorded from 360–600 nm. For CR binding assay, incubated aliquots of bovine insulin (final concentration of 5 μM) or HEWL (final concentration of 2.5 μM) solutions, were added to 950 μl of CR solution (20 μM). After 30 min of incubation at RT, absorbance spectra were recorded between 400 and 600 nm. The effect of GOQDs/rGOQDs on the secondary structural changes of bovine insulin, incubated under amyloidogenic conditions, was investigated using far-UV CD spectroscopy. Briefly, aliquots of incubated samples were removed and diluted to a final concentration of 50 μM. The spectra were recorded in the range of 190–260 nm using a AVIV 215 spectropolarimeter (Aviv Associates, Lakewood, NJ, USA). Finally, for AFM imaging, aliquots of incubated bovine insulin or HEWL solutions were removed and diluted 50 and 10 fold with DW, respectively. Then, 10 μl of diluted sample was placed on freshly cleaved mica and dried at RT. Images were acquired in non-contact mode using a quantitative AFM (ARA-AFM, Ara-Research Company, Iran).

### Cell toxicity assay

Human neuroblastoma SH-SY5Y cells, were cultured in DMEM-F12 medium as reported previously [42]. For cytotoxicity experiments, protein samples aged alone or with various concentrations of GOQDs or rGOQDs under amyloidogenic conditions were added to the cells (final concentration of 10 μM) and left for 24 h. Cells treated with H_2_O/HCl solution (pH 1.5) or 50 mM glycine buffer (pH 2.2) were used as control. Then, 10 μl of MTT stock solutions (5 mg/ml in PBS) were added to 100 μl of DMEM-F12 containing 10% FBS, followed by incubation for 3 h. Solutions were aspirated, and cells were treated with DMSO for 15 min, followed by absorbance measurement at 570 nm using an ELISA reader. Results were expressed as percentage of MTT reduction relative to the control cells, assuming that the absorbance of the control was 100%.

### Statistical analysis

All assays were performed two or three times with triplicate repeats. Data are expressed as percentage of values in untreated control cells, with each value representing the mean ± SD (n = 3). The significant differences between the means of the treated and untreated groups were calculated by unpaired Student's t-test and p-values less than 0.05 were considered significant. *p < 0.05; **p < 0.01, were significantly different from control. ^#^p < 0.05; ^##^p < 0.01, were significantly different from those exposed only to amyloid fibrils.

## Result and discussion

### Characterization of GOQDs and rGOQDs

**Fig 2:**
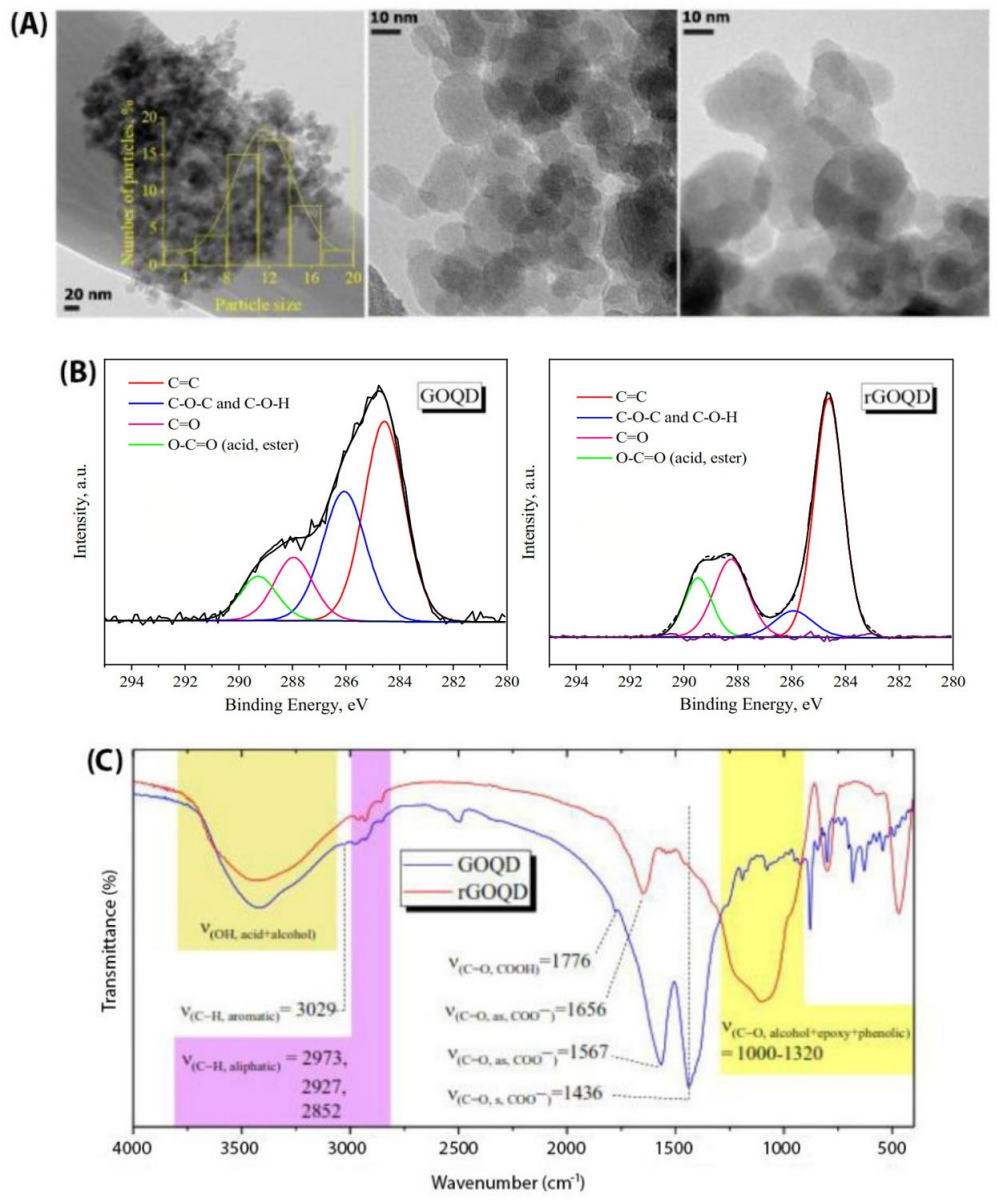
Microscopic and spectroscopic characterizations of GOQDs and rGOQDs. (A) TEM images of 0.02 mg/ml GOQDs at different magnifications (inset shows the size distribution of GOQDs); (B) The XPS C1s spectra of GOQDs and rGOQDs, and (C) FT-IR spectra of KBr pellets of GOQDs and rGOQDs.

TEM images revealed a circular sheet morphology for GOQDs with a narrow size distribution ranging from 2 to 20 nm and a number average size of 11.4 nm (Fig 2A), while large aggregates of rGOQDs were observed due to a reduction in the number of interacting functional groups on the surface of rGOQDs (Fig A in S1 File).

**Fig 3:**
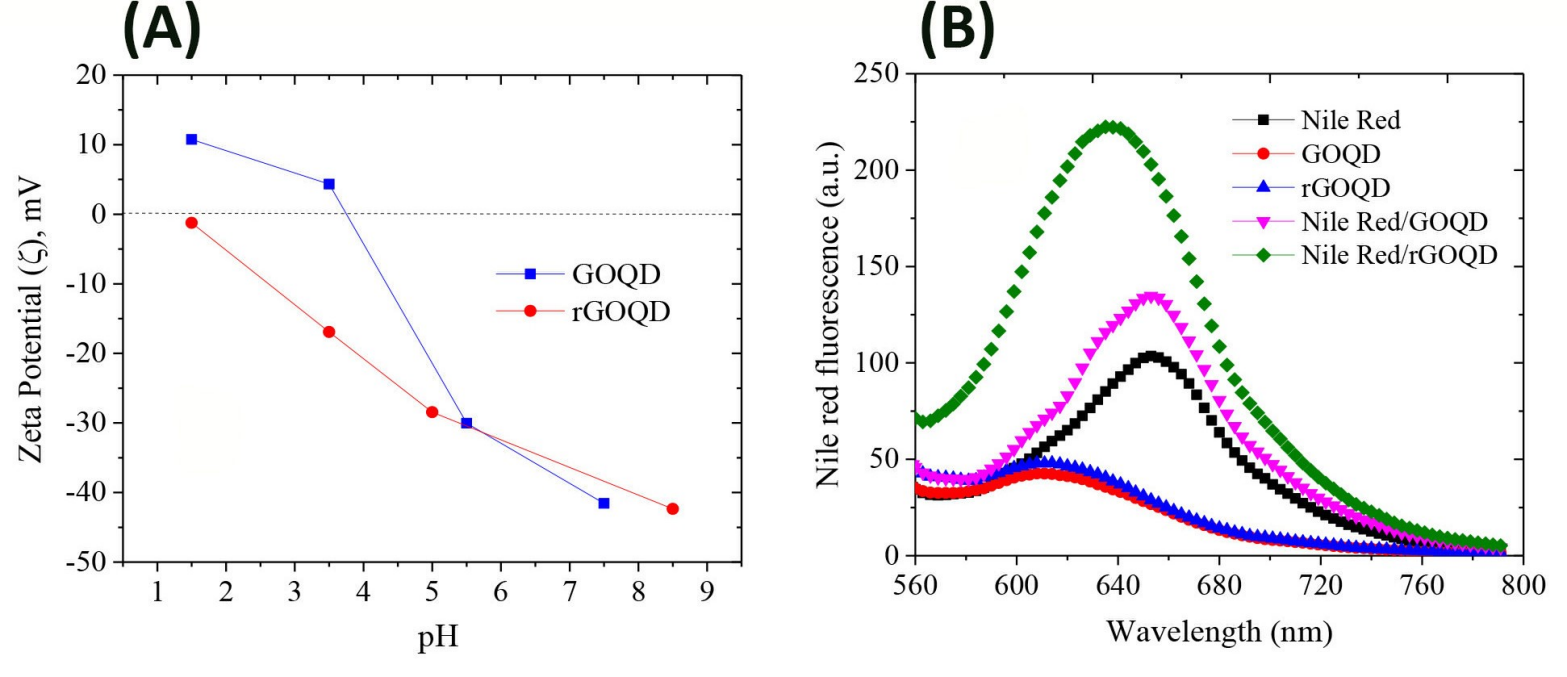
Zeta potential and surface hydrophobicity measurements of GOQDs and rGOQDs. (A) Zeta potential changes of 0.2 mg/ml GOQDs and rGOQDs in a range of pH (1.5–8.5); (B) Fluorescence intensity of Nile red in the absence and presence of 2 mg/ml GOQDs and rGOQDs. The fluorescence spectra of GOQDs and rGOQDs excited at 530 nm are also shown.

In order to determine the chemical composition of GOQDs and rGOQDs, XPS analysis was performed (Fig 2B). Both particles display the C and O in the survey XPS spectra. The curve fitting of the high-resolution spectrum of C yields major peak at 284.5 eV corresponding to C = C sp^2^ carbon and three peaks in the range of 285–290 eV related to C-O, C = O and O-C = O groups, respectively. Quantitative analysis of the XPS peaks indicate that the fraction of C = C graphitized carbon decreased from 59% for GOQDs to 39% for rGOQDs (Fig 2B). One can conclude that the oxygen-containing groups on the surface of rGOQDs are less than those in GOQDs, confirming successful reduction of GOQDs. The UV-Vis absorption and FT-IR spectroscopy were used to further confirm reduction of GOQDs. In the FT-IR spectra of GOQDs, stretching vibration for carbonyl group in carboxylic acid (1776 cm^-1^), symmetric and asymmetric stretching vibrations of carbonyl group in carboxylate (1436 and 1567 cm^-1^, respectively), aliphatic C-H stretching vibration (2852, 2927, 2973 cm^-1^), aromatic C-H stretching vibration (3029 cm^-1^), C-O stretching vibration of ether, phenol and ester groups (1000–1320 cm^-1^) and O-H stretching vibration of hydroxyl and carboxylic acid (3000–3600 cm^-1^) were observed (Fig 2C). For rGOQDs, intensity of peaks related to the hydroxyl and all carbonyl groups was decreased or disappeared, while the intensity of peak related to the C-O-C group was increased (Fig 2C). Moreover, surface structural changes upon chemical reduction led to a decrease in UV absorption, along with a blue shift (Fig B in S1 File) [29, 44, 45]. As shown in Fig 3A, zeta potential of GOQDs was decreased gradually with change in pH from +10.7 to -41.5 mV, suggesting an isoelectric point around 4.

The rGOQDs showed a negative zeta potential in the whole pH range and decreased gradually with pH (Fig 3A). It has been suggested that the positive surface charge of carbon dot materials, including graphene quantum dots, may be the result of two possible mechanisms: I) protonation of oxygen-containing functional groups acting as brønsted base and II) proton complexation of the π–electron systems of graphene planes (sp^2^ aromatic hybridization; C_π_+H_2_O→ C_π_H^+^ + OH^-^). Their negative surface charge, however, may be attributed to deprotonation of phenolic, enolic and carboxylic groups [46, 47]. Since the oxygen-containing groups disappear during the reduction process, rGOQDs show a negative surface charge in different pH ranges (Fig 3A). Generally, the negatively charged colloids with zeta potential more than -30 mV form electrostatically stable and well-dispersed suspensions in aqueous solutions [48–50]. Despite high zeta potential values for both GOQDs and rGOQDs (more than -30 mV) at pH 7 (Fig 3A), some precipitation was observed for the rGOQDs aqueous dispersions. A simple explanation for this observation may be particles aggregation which can be attributed to the hydrophobic nature of rGOQDs, π-π stacking of rGOQDs sheets, and finally increased attractive Van der Waals interactions [51–53]. Nile red is a hydrophobic dye whose fluorescence maximum strongly increases along with a blue shift upon interaction with hydrophobic surfaces [54]. As shown in Fig 3B, in the presence of GOQDs, only a slight increase in Nile red fluorescence intensity was observed without any shift to lower wavelengths. On the other hand, incubation of Nile red with rGOQDs led to a significant enhancement in its fluorescence, with a pronounced blue shift, from 654 nm to 635 nm (Fig 3B), indicating increased surface hydrophobicity of GOQDs upon the reduction process. This may be attributed to partial remove of oxygen-containing carboxylic, hydroxyl, and epoxy groups presented on the surface of GOQDs. This increased hydrophobicity of rGOQDs (Fig 3B) may promote surface interactions between two or more rGOQDs, leading to the formation of large aggregates, as evidenced by AFM (Fig C in S1 File).

### Effect of GOQDs/rGOQDs on bovine insulin amyloid fibril formation

**Fig 4:**
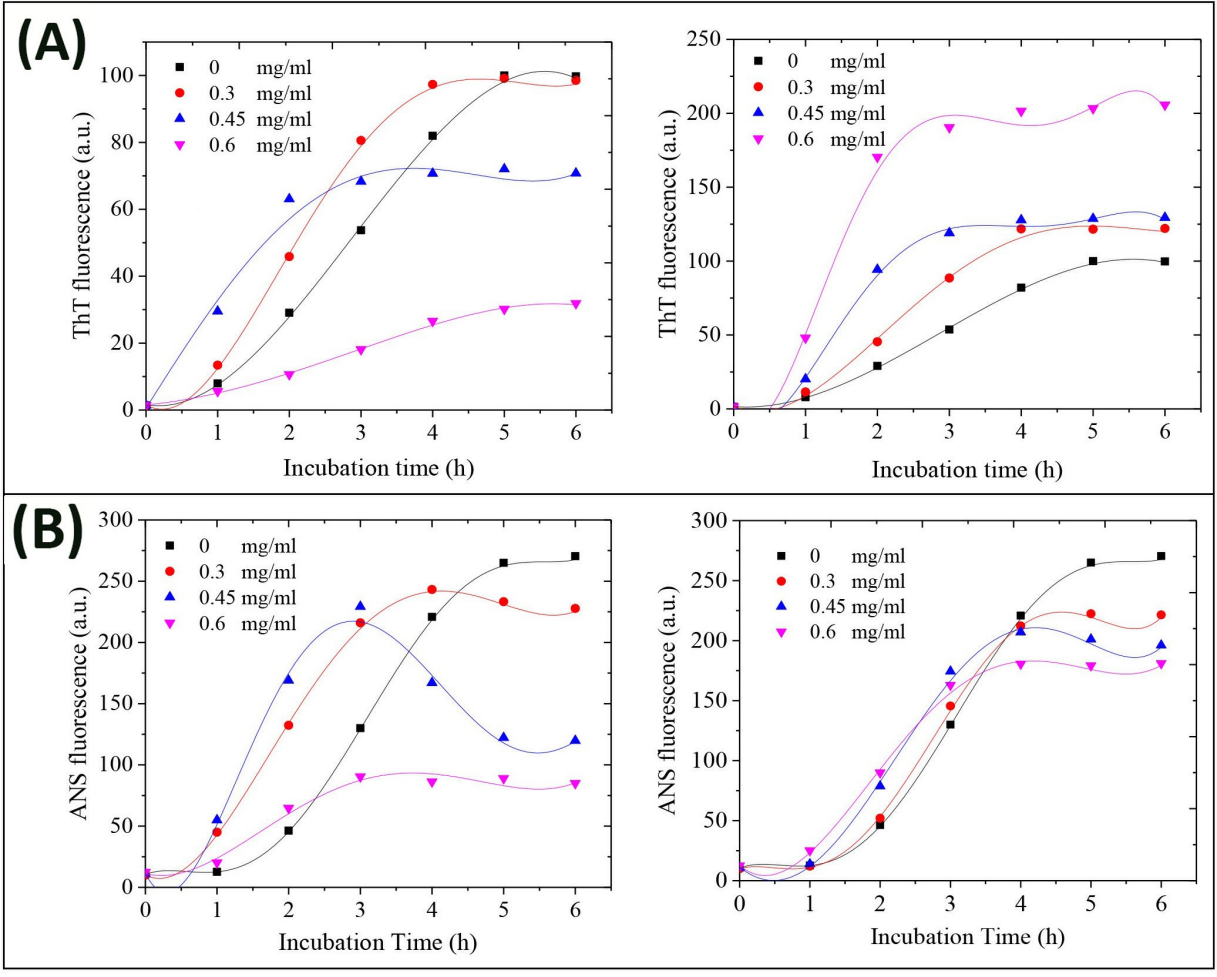
Effect of GOQDs and rGOQDs on the kinetics of amyloid fibrillation (A) and surface hydrophobicity (B) of bovine insulin as monitored by ThT and ANS fluorescence assays, respectively. Protein samples (100 μM) were incubated in H_2_O/HCl solution (pH 1.5) at 57 ^○^C either alone or with various concentrations of GOQDs (left graphs) or rGOQDs (right graphs) and fluorescence intensity was measured for 6 h. Each experiment was performed in triplicate.

Time course, as well as lag phase duration of bovine insulin amyloid fibrillation without or with various concentrations of GOQDs or rGOQDs was monitored by fluorescence measurement of ThT, an amyloid-specific dye, which it’s binding with amyloidogenic species is directly correlated with their β-sheet content [55]. Although, co-incubation with GOQDs resulted in a significant and dose-dependent decrease in the final intensity of ThT fluorescence, the effect of different concentrations of GOQDs on the kinetics of insulin amyloid fibrillation was not dose-dependent (Fig 4A, left graph).

While in the presence of 0.3 and 0.45 mg/ml GOQDs a concentration-dependent decrease in the nucleation phase was observed, it was significantly prolonged in samples incubated with 0.6 mg/ml GOQDs (Fig 4A, left graph and Table 1).

**Table 1:** Effect of GOQDs/rGOQDs on the kinetics parameters of bovine insulin fibrillization determined by ThT fluorescence assay.

	Lag time (h)	K_app_ (h^-1^)	Amplitude (a.u.)
Bovine insulin	1.00 ± 0.09	1.00 ± 0.02	99.76 ± 6.43
**GOQDs**			
0.3 mg	0.90 ± 0.04	1.19 ± 0.03	98.51 ± 4.09
0.45 mg	0.2 ± 0.01	1.28 ± 0.01	70.75 ± 6.31
0.6 mg	1.1 ± 0.06	0.29 ± 0.01	31.89 ± 2.33
**rGOQDs**			
0.3 mg	0.80 ± 0.05	1.56 ± 0.42	122.04 ± 8.99
0.45 mg	0.52 ± 0.07	2.06 ± 0.31	129.42 ± 5.43
0.6 mg	0.31 ± 0.03	3.52 ± 0.82	202.73 ± 12.76

**Fig 5:**
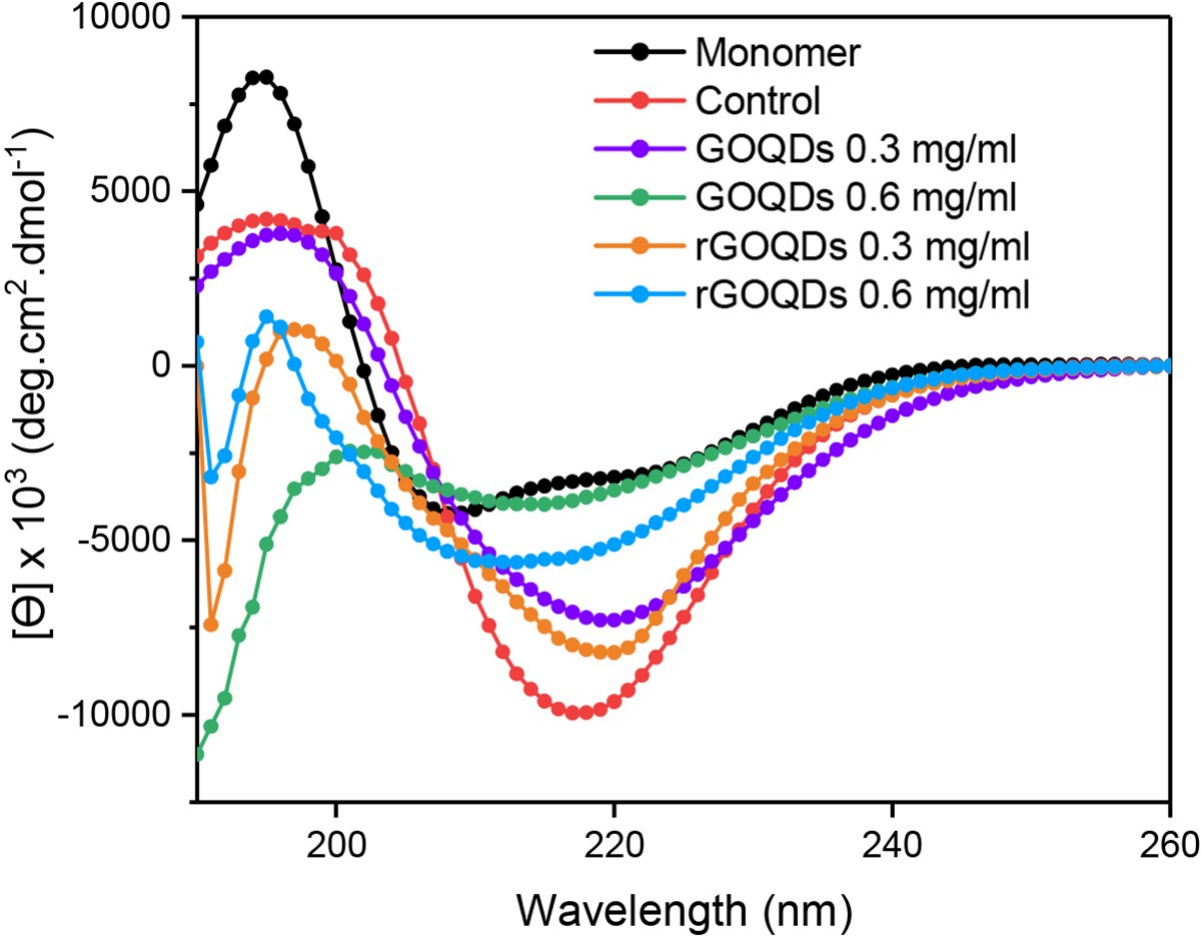
Effect of GOQDs and rGOQDs on the secondary structural changes of bovine insulin incubated under amyloidogenic conditions. Further details are described in Material and methods section.

This finding suggests that the effect of GOQDs on the kinetics of insulin fibrillation may be dependent on protein:nanoparticle molar ratios. In accord with this observation, Cabaleiro-Lago et al. reported acceleration (by shortening of the lag phase) and retardation (by prolonging of the lag phase) of amyloid β fibrillation, at low and high concentrations of nanoparticles, respectively [56]. On the contrary, for samples incubated with varied concentrations of rGOQDs, a dose-dependent increase in the final intensity of ThT fluorescence was observed, along with shortening of the nucleation phase (Fig 4A, right graph and Table 1), suggesting that promotion of insulin aggregation may be a mechanistic feature of their effect. A possible explanation for this may be related to the enhanced surface hydrophobicity of rGOQDs, induced by the reduction process (Fig 3B), which can promote the assembly process. These data are in accord with earlier reports demonstrating promotion of fibrillation of different peptides and proteins, in the presence of carbon nanoparticle derivatives [57–59]. Taken together, we propose that any difference in the mechanism of action between GOQDs and rGOQDs may be attributed to their surface structural and morphological features (such as hydrophobicity, charge, and size) which appear to play a critical role in nanoparticle-protein interactions [59]. While a dose-dependent decrease in CR absorbance was observed in samples incubated with GOQDs, the presence of rGOQDs caused no significant change (Fig D in S1 File), further confirming a structure-dependent mechanism. It is well-established that under amyloidogenic conditions, various proteins, including bovine insulin and HEWL, undergo conformational changes characterized by solvent-exposure of hydrophobic regions [60–62]. The effect of GOQDs on the conformational changes of bovine insulin in the course of fibrillation process was monitored by ANS fluorescence measurement, demonstrating a pattern similar to ThT fluorescence (Fig 4B, left graph). For samples treated with rGOQDs, despite a dose-dependent increase in ThT fluorescence (Fig 4A, right graph), ANS emission intensity was slightly decreased dose-dependently (Fig 4B, right graph). This contradiction may be attributed to the binding of ThT to β-sheets as a common structural element in both amyloid fibrils and amorphous aggregates [63–65], indicating that structures produced in the presence of rGOQDs contain high quantities of β-sheets with reduced solvent-exposed hydrophobic patches. To elucidate the secondary structural transitions of bovine insulin induced by GOQDs/rGOQDs, far-UV CD spectroscopy was applied. For samples incubated alone, a gradual transition from a predominantly α-helical to a β-sheet-rich structure, characterized by a negative peak at 218 nm, confirmed the formation of amyloid fibrils (Fig 5).

**Fig 6:**
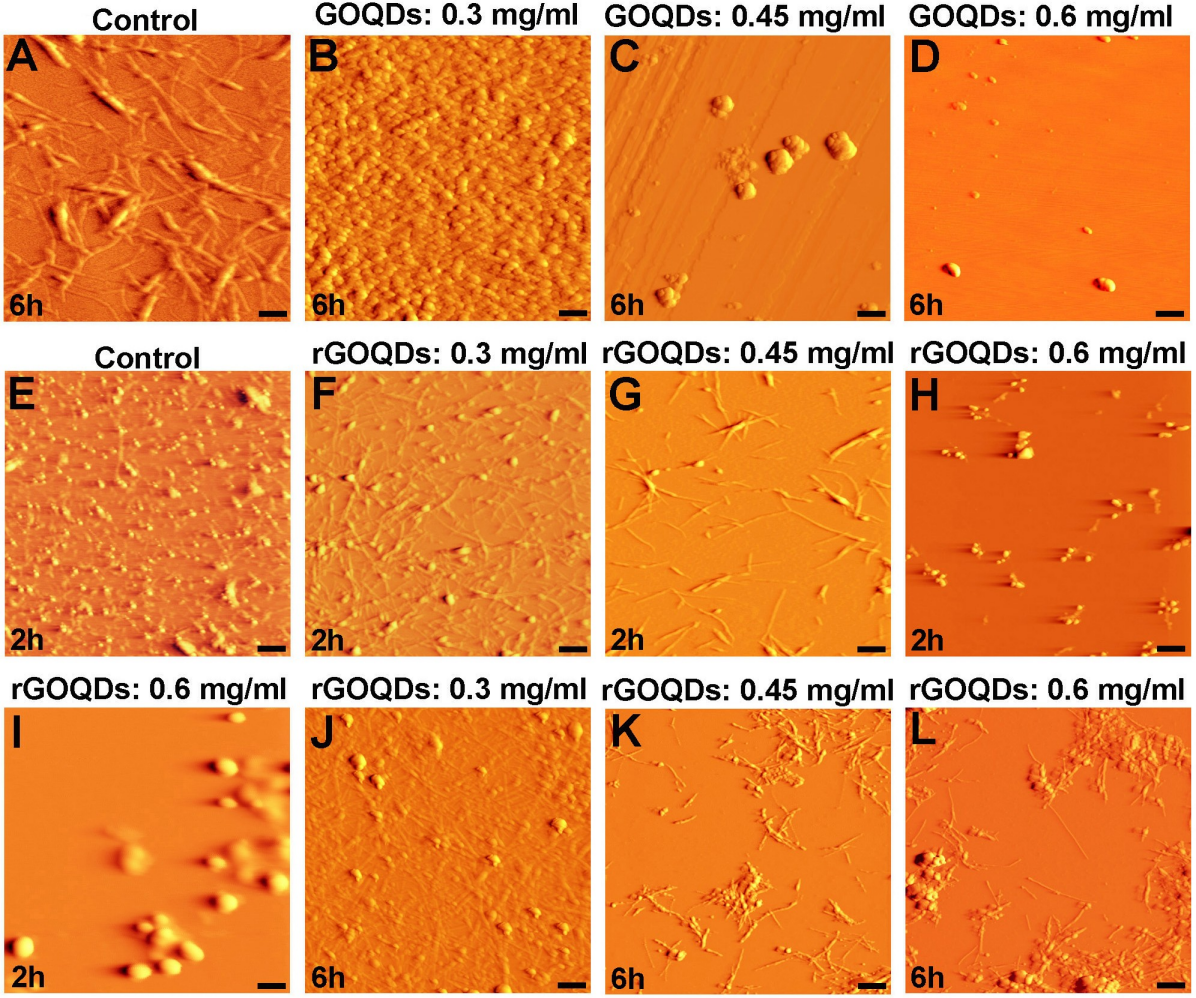
AFM images of bovine insulin incubated with different concentrations of GOQDs or rGOQDs. Protein samples (100 μM) were incubated in H_2_O/HCl solution (pH 1.5) at 57 ^○^C either alone or with various concentrations of GOQDs or rGOQDs. (I) An enlarged view of (H). The scale bar is 100 nm in (I) and 500 nm in other images.

While appearance of the negative peak was dose-dependently hindered in the presence of both GOQDs and rGOQDs, GOQDs appeared to be more effective (Fig 5). This data suggests that the structures produced in the presence of GOQDs contain lower quantities of β-sheets, which is in accord with ThT results (Fig 4A). Finally, to further corroborate the above observations, AFM imaging was employed (Fig 6).

Inhibitory effects of GOQDs on bovine insulin fibrillation was seen to follow a concentration-dependent pattern (Fig 6B–6D), consistent with the ThT, ANS, CR, and CD results. On the other hand, treatment of protein samples with rGOQDs appear to have resulted in the acceleration of insulin aggregation. To directly observe and further confirm this proposal, the AFM images of samples withdrawn at different times of 2 and 6 h after incubation (corresponding to the beginning of growth phase and the plateau phase, respectively) were acquired. As shown in Fig 6E, for insulin alone, oligomeric structures were predominantly apparent after 2 h incubation. On the other hand, in the presence of 0.3 mg/ml rGOQDs, oligomeric species with the appearance of small protofibrils were observed (Fig 6F). In the presence of 0.45 mg/ml rGOQDs, however, short fibrils appeared (Fig 6G). In samples containing 0.6 mg/ml rGOQDs, the amount of oligomers decreased significantly and instead some amorphous aggregates became obvious (Fig 6H and 6I, and Fig E in S1 File), suggesting that rGOQDs have the capacity of influencing the early stages of insulin fibrillation, leading to a significant loss of oligomers, in accord with ThT results (Fig 4A, right graph and Table 1). The ability of rGOQDs to accelerate insulin aggregation was better confirmed using AFM images taken after 6 h of incubation. As shown in Fig 6J, in samples incubated with 0.3 mg/ml rGOQDs, formation of such amyloid fibrils was prominently inhibited, with the appearance of protofibrillar structures and some amorphous aggregates. In the presence of 0.45 mg/ml rGOQDs, bundles of short fibrils were observed (Fig 6K) followed by the appearance of a combination of short fibrils and amorphous aggregates, assembled in very large structures (Fig 6L and Fig E in S1 File). Taken together, these findings suggest that GOQDs may exert their inhibitory effects by preventing amyloid fibril formation and/or redirecting the insulin assembly process toward an alternative disordered (amorphous) aggregation pathway. On the other hand, rGOQDs may promote insulin assembly via shortening of the nucleation phase and directing aggregation toward formation of large amorphous aggregates bundled with small fibrils.

### Effect of GOQDs/rGOQDs on HEWL amyloid fibril formation

**Fig 7:**
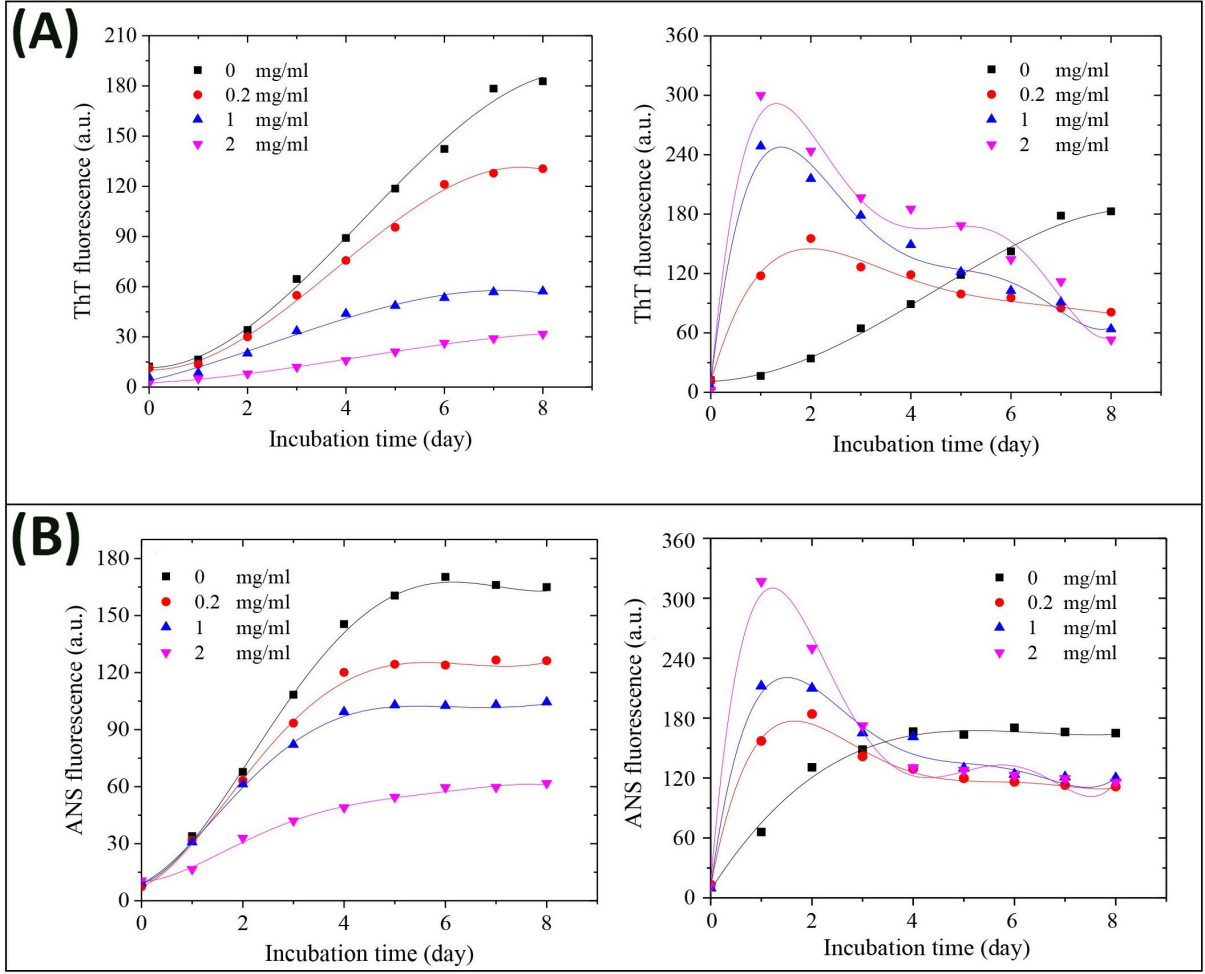
Effect of GOQDs and rGOQDs on the kinetics of amyloid fibrillation (A) and surface hydrophobicity (B) of HEWL as monitored by ThT and ANS fluorescence assays, respectively. Protein samples (50 μM) were incubated in 50 mM glycine (pH 2.2) at 57 ^○^C either alone or with various concentrations of GOQDs (left graphs) or rGOQDs (right graphs) and fluorescence intensity was measured for 8 days. Each experiment was performed in triplicate.

Kinetics of HEWL amyloid fibril formation alone or in the presence of different molar ratios of GOQDs and rGOQDs are shown in Fig 7.

Obtained results indicate a concentration-dependent decrease in ThT fluorescence intensity, along with prolongation of the nucleation phase, for samples incubated with GOQDs (Fig 7A, left graph and Table 2).

**Table 2:** Effect of GOQDs/rGOQDs on the kinetics parameters of HEWL fibrillization determined by ThT fluorescence assay.

	Lag time (h)	K_app_ (h^-1^)	Amplitude (a.u.)
**HEWL**	21.6 ± 2.03	1.17 ± 0.04	182.75 ± 12.43
**GOQDs**			
0.2 mg	24.2 ± 1.87	0.89 ± 0.02	130.5 ± 8.65
1 mg	26.4 ± 1.53	0.49 ± 0.02	57.25 ± 5.03
2 mg	-^a^	0.18 ± 0.1	31.75 ± 1.01
**rGOQDs**			
0.2 mg	-^b^	3 ± 0.42	80.92 ± 6.91
1 mg	-^b^	10.12 ± 4.98	63.92 ± 5.49
2 mg	-^b^	12.41 ± 3.65	53.11 ± 2.54

^a^ The lag time could not be determined since aggregation was not observed.

^b^ The lag time could not be determined since aggregation was very fast.

**Fig 8:**
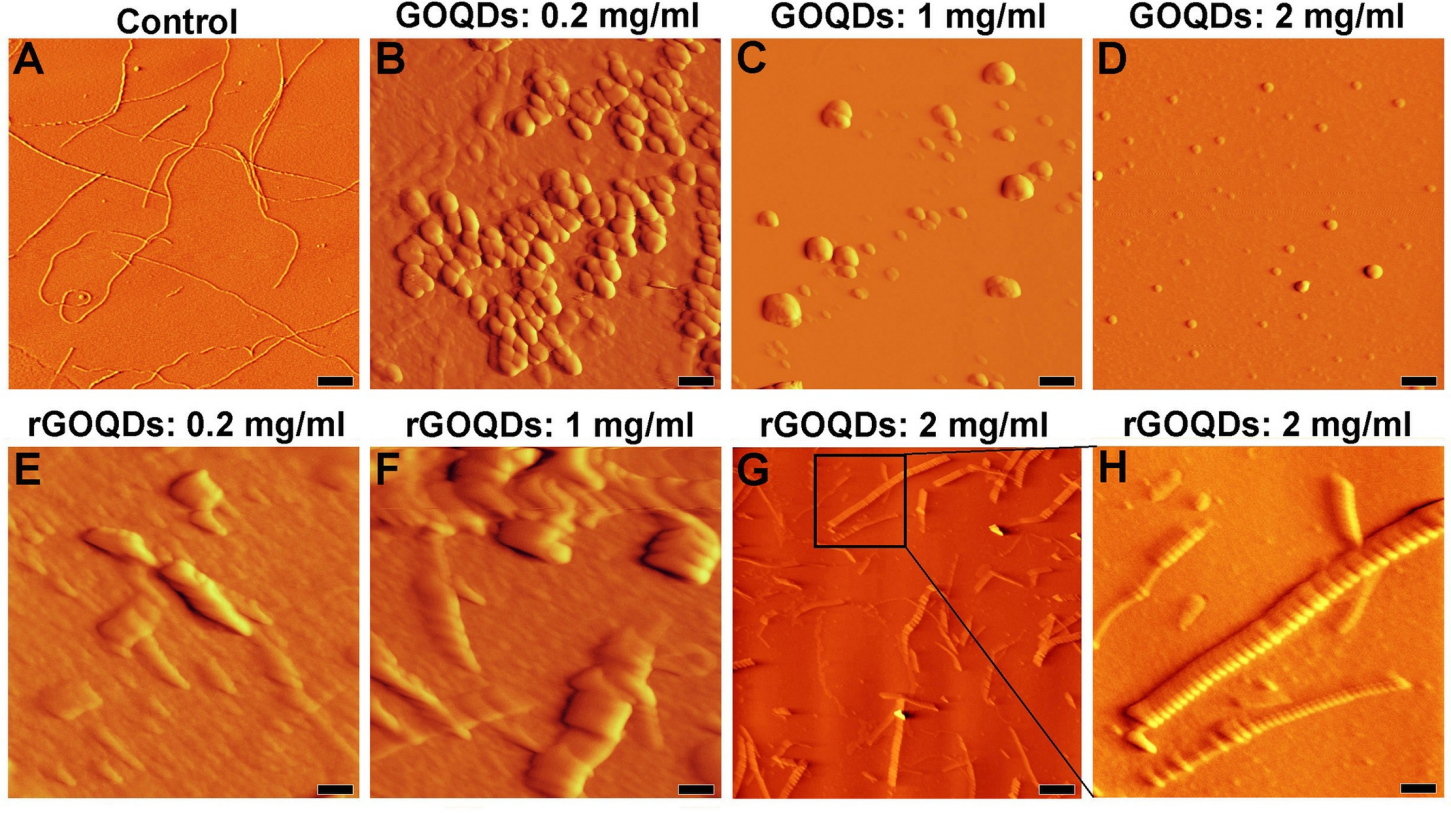
AFM images of HEWL incubated with different concentrations of GOQDs or rGOQDs. Protein samples (50 μM) were incubated in 50 mM glycine (pH 2.2) at 57 ^○^C either alone or with different concentrations of GOQDs, or rGOQDs. (H) An enlarged view of (G). The scale bar is 2000 nm in (G) and 500 nm in the other images.

In the presence of rGOQDs, however, we observed a rapid and dose-dependent increase in ThT fluorescence intensity, followed by a significant decrease (Fig 7A, right graph and Table 2), presumably due to lateral fibril-fibril interactions, leading to eventual burial of β-sheets in a more compact, less available structure for ThT binding. This finding indicates that shortening of the nucleation phase may be the mechanism by which rGOQDs affect the aggregation process (Table 2). While the presence of GOQDs effectively prevented CR absorbance enhancement, a marked and dose-dependent increase in CR absorbance was observed for samples incubated with rGOQDs (Fig F in S1 File). A significant and dose-dependent decrease in ANS fluorescence intensity (Fig 7B, left graph) suggested that blocking of hydrophobic patches may be one of the mechanisms by which GOQDs inhibit HEWL amyloid fibrillation, in accord with an earlier report [66]. On the other hand, in the presence of rGOQDs, ANS fluorescence intensity was greatly increased at initial incubation times, followed by a significant decrease (Fig 7B, right graph), similar to fluorescence emission pattern of ThT (Fig 7A, right graph) likely due to lateral fibril-fibril interactions. Morphology of HEWL aggregates produced alone, or in the presence of GOQDs or rGOQDs, were analyzed by AFM (Fig 8).

As illustrated in Figs 8B and 7D, the inhibitory effect of GOQDs on HEWL fibrillation follows a concentration-dependent pattern, which is consistent with the results obtained by ThT, ANS, and CR assays (Fig 7A and 7B (left graphs), and Fig F in S1 File). On the contrary, the presence of rGOQDs was not only ineffective in preventing HEWL amyloid fibrillation, but (dose-dependently) promoted the assembly of HEWL (Fig 8E–8H). In the presence of the highest concentration of rGOQDs, very large fibrillar structures with an average diameter of 500 nm were observed (Fig 8G and 8H). For samples incubated with rGOQDs, while obtained results showed a decrease in ThT and ANS fluorescence intensity (Fig 7A and 7B, right graphs), a significant enhancement in CR absorbance (Fig F in S1 File), together with the appearance of very large fibrillar structures were indicated (Fig 8G and 8H). A simple explanation for this observation could be that promotion of HEWL amyloid fibrillation by rGOQDs may result in the formation of very large amyloid fibrils with a more compact, less flexible structures, leading to the loss of their ability to bind ThT and ANS [60]. Based on the results obtained by ThT, ANS, CR, and AFM, it appears that while GOQDs are effective in inhibiting HEWL amyloid fibrillation, rGOQDs may promote protein assembly.

### Effects of GOQDs/rGOQDs on cytotoxicity of bovine insulin and HEWL aggregates

**Fig 9:**
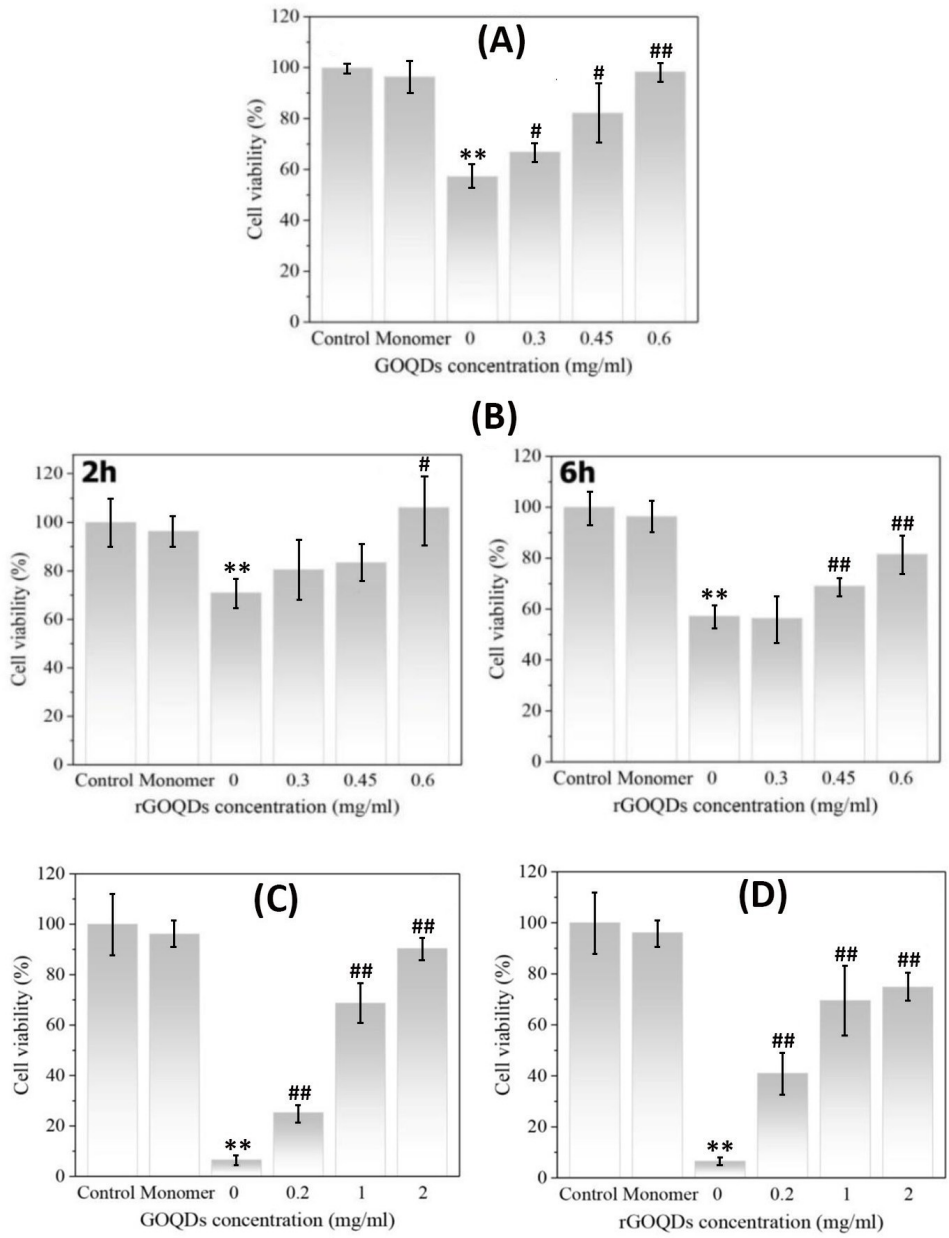
Protective effect of GOQDs and rGOQDs against cytotoxicity induced by bovine insulin and HEWL aggregates measured by MTT assay. For bovine insulin, SH-SY5Y cells were treated with 6-h-old insulin aggregates (10 μM) aged alone, or in the presence of various concentrations of GOQDs (A); or 2-hours-old and 6-hours-old insulin aggregates (10 μM) aged alone or in the presence of various concentrations of rGOQDs (B). For HEWL, SH-SY5Y cells were treated with HEWL aggregates (10 μM) aged alone or in the presence of various concentrations of GOQDs (C) or rGOQDs (D). The data are expressed as percentage of values in untreated control cells. Each value represents the mean ± SD (n = 3). *p < 0.05;**p < 0.01, were significantly different from control cells. ^#^p < 0.05; ^##^p < 0.01, were significantly different from cells exposed only to bovine insulin/HEWL amyloid fibrils.

Finally, toxicity of bovine insulin and HEWL aggregates produced in the presence of GOQDs or rGOQDs was examined using MTT assay. The nontoxic doses of both GOQDs and rGOQDs were determined based on the viability of SH-SY5Y cells. We found that cells exposed to different concentrations of GOQDs or rGOQDs (0.2–2 mg/ml) for 24 h experienced no significant toxicity (Fig G in S1 File). Based on our recent report [67], a 10 μM bovine insulin fibril preparation was used to induce SH-SY5Y cell injury. As shown in Fig 9A, the presence of GOQDs dose-dependently increased SH-SY5Y viability as in the presence of 0.6 mg/ml we observed a significant increase of 98.33 ± 3.49 (relative to control cells assuming 100% cell viability) on cell survival.

To test toxicity of structures produced in the presence of rGOQDs, cells were exposed to samples incubated for 2 and 6 h under amyloidogenic conditions. The presence of 2-hours-old insulin aggregates, aged alone, led to a decrease of 29.05 ± 5.96 (relative to control cells assuming 100% cell viability) on cell survival (Fig 9B, left graph), presumably due to the presence of oligomeric species (Fig 6E). However, the presence of rGOQDs resulted in a dose-dependent enhancement of cell viability (Fig 9B, left graph). This increased rescue was observed, to a lesser extent, for samples incubated for 6 h (Fig 9B, right graph), presumably due to the presence of short amyloid fibrils (Fig 6J–6L). For HEWL aggregates, the presence of GOQDs and rGOQDs dose-dependently increased SH-SY5Y cells survival, indicating that the aggregates are significantly nontoxic (Fig 9C and 9D). Overall, obtained results suggest that aggregated species produced in the presence of GOQDs/rGOQDs show reduced toxicity.

## Concluding remarks

**Fig 10:**
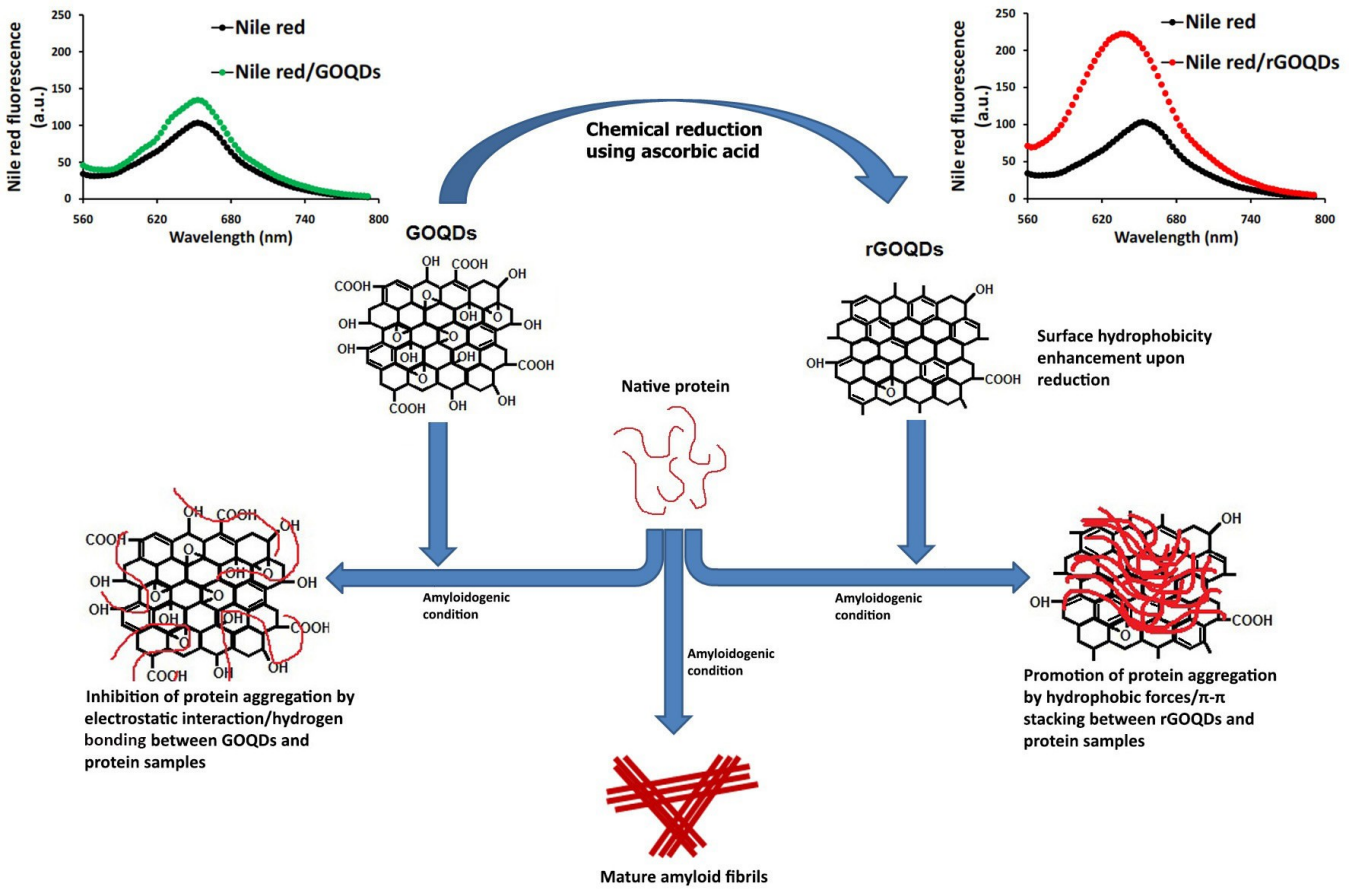
Schematic presentation of possible mechanism of GOQDs/rGOQDs action on modulating amyloid fibril formation.

Results show the capacity of both GOQDs and rGOQDs to modulate the assembly of the two tested proteins, but through different mechanisms. The inhibitory effects of GOQDs may be attributed to electrostatic interactions and/or hydrogen bonding between GOQDs and protein molecules, which are mediated by oxygen-containing functional groups, presented at the surface of GOQDs. We suggest that these interactions would stabilize protein structure, and thereby prevent further conformational changes, leading to inhibition of amyloid fibril formation (Fig 10).

Unlike amyloid fibrillation inhibition by GOQDs, the presence of rGOQDs appear to promote assembly of both proteins via shortening the nucleation phase. Clearly, reduction with AA would remove some of the oxygen-containing functional groups from the GOQDs surface. This may lead to a decrease in the occurrence of electrostatic interactions/hydrogen bonding between rGOQDs surface and proteins, as well as surface hydrophobicity enhancement of rGOQDs. We postulate that the increased surface hydrophobicity may provide rGOQDs with the capacity to interact with partially unfolded species, and promote their assembly toward formation of large amorph aggregates (Fig 10). However, it is noteworthy that, low water solubility of rGOQDs and their propensity for precipitation may limit their *in vivo* applications. One of the most promising approaches to overcome this problem is nanonization strategies, commonly used for poorly water-soluble drugs [68]. Although further studies are needed to elucidate the precise mechanism of action, it appears that both GOQDs and rGOQDs have the potential to be considered as non-toxic amyloid fibrillation inhibitors in neurological diseases.

## Supporting information

S1 File Fig A. TEM images of rGOQDs acquired from different preparations. Fig B. UV−Vis absorption of 0.3 mg/ml of GOQDs and rGOQDs aqueous solutions. Fig C. AFM and 3D topographical images of GOQDs **(A)** and rGOQDs **(B)** and related line profiles **(C)**. **(D)** An enlarged view of GOQDs line profile. The arrows in (B) indicate some aggregates formed upon reduction. The scale bars represent 500 nm. Fig D. Congo red binding absorption spectra of bovine insulin in the absence and presence of various concentrations of GOQDs **(A)** and rGOQDs **(B)**. Congo red absorbance alone and in the presence of native bovine insulin are also indicated. Fig E. Line profiles of AFM images relating to Fig 4H, 4I and 4L. Fig F. Congo red binding absorption spectra of HEWL in the absence and presence of various concentrations of GOQDs **(A)** and rGOQDs **(B)**. Congo red absorbance alone and in the presence of native HEWL are also indicated. Fig G. Effect of various concentrations of GOQDs and rGOQDs on the viability of SH-SY5Y cells.
(DOCX)
