# Supplementary material for: Role of surface oxygen-containing functional groups of graphene oxide quantum dots on amyloid fibrillation of two model proteins
Source: PLoS One. 2020 Dec 23;15(12):e0244296. doi: 10.1371/journal.pone.0244296 (PMC7757872; doi:10.1371/journal.pone.0244296)

**Role of surface oxygen-containing functional groups of graphene oxide quantum dots on amyloid fibrillation of two model proteins**

Ebrahim Rostampour Ghareghozloo^1^, Mohsen Mahdavimehr^1^, Ali Akbar Meratan^1*^, Nasser Nikfarjam^2^, Atiyeh Ghasemi^3^, Bentolhoda Katebi^1^, Mohsen Nemat-Gorgani^4^

^1^Department of Biological Sciences, Institute for Advanced Studies in Basic Sciences (IASBS), Zanjan, Iran

^2^Department of Chemistry, Institute for Advanced Studies in Basic Sciences (IASBS), Zanjan, Iran

^3^Institute of Biochemistry and Biophysics, University of Tehran, Tehran, Iran

^4^Stanford Genome Technology Center, Stanford University, Palo Alto, CA, USA

**S1 File Fig A.** TEM images of rGOQDs acquired from different preparations .

**
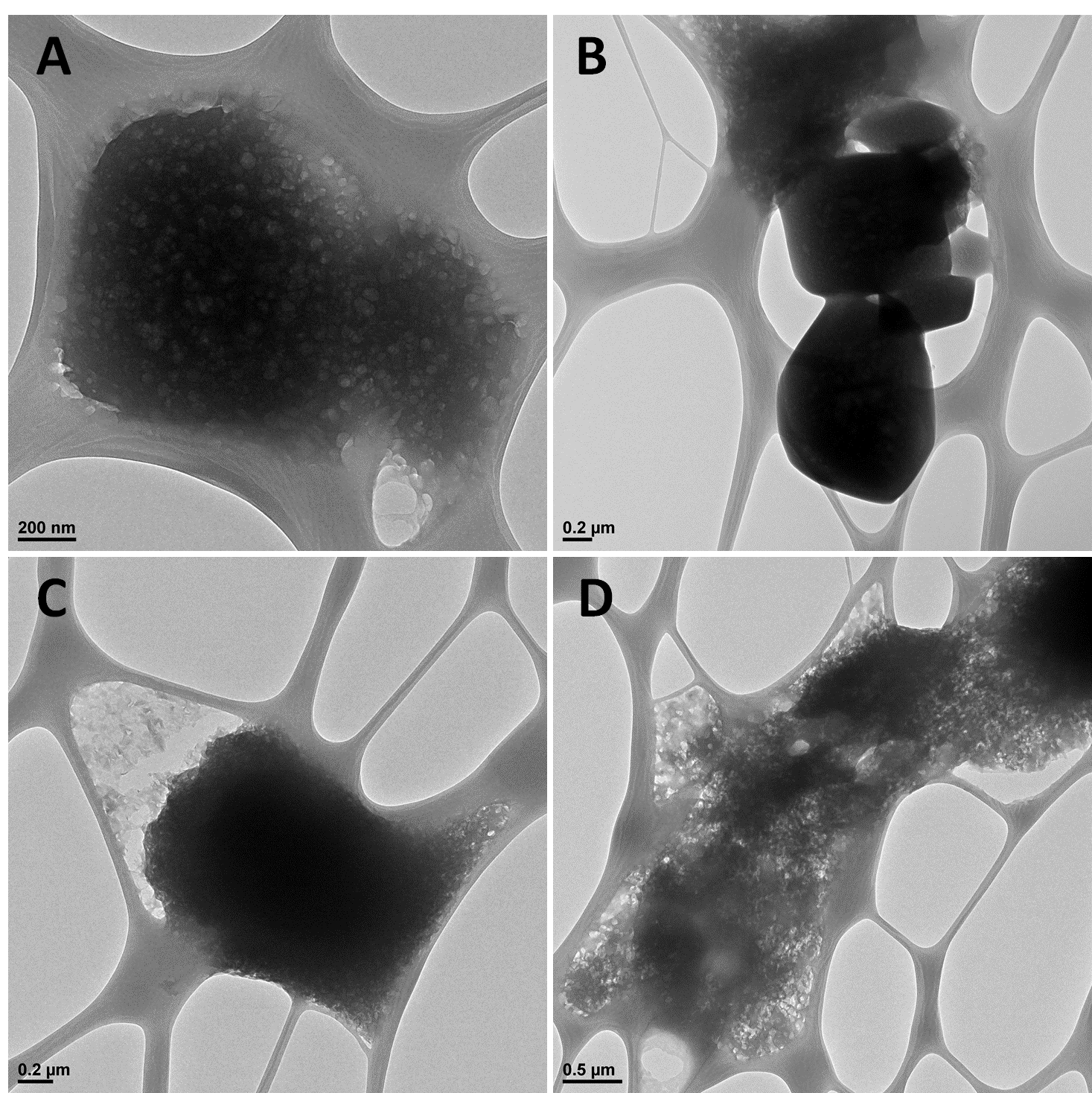
**

**S1 File Fig B.** UV−Vis absorption of 0.3 mg/ml of GOQDs and rGOQDs aqueous solutions.


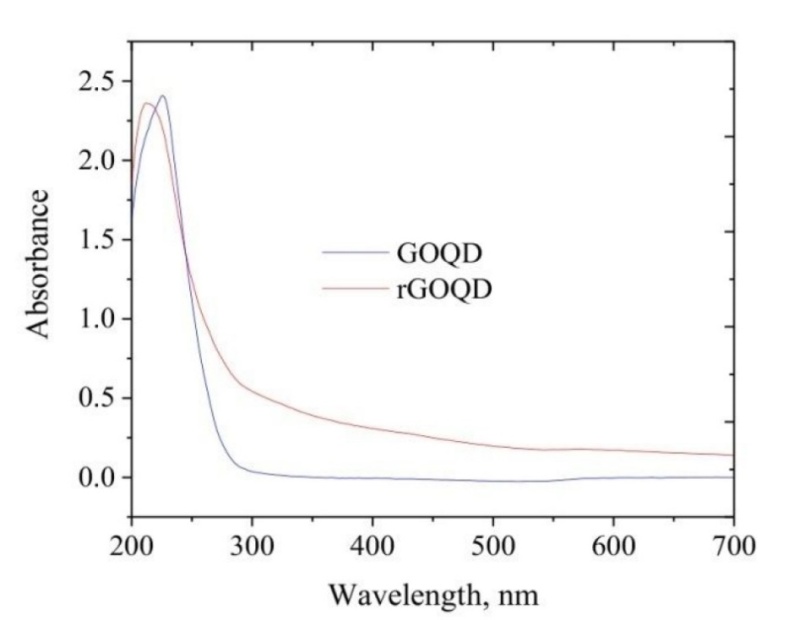


**S1 File Fig C.** AFM and 3D topographical images of GOQDs (A) and rGOQDs (B) and related line profiles (C). (D) An enlarged view of GOQDs line profile. The arrows in (B) indicate some aggregates formed upon reduction. The scale bars represent 500 nm.


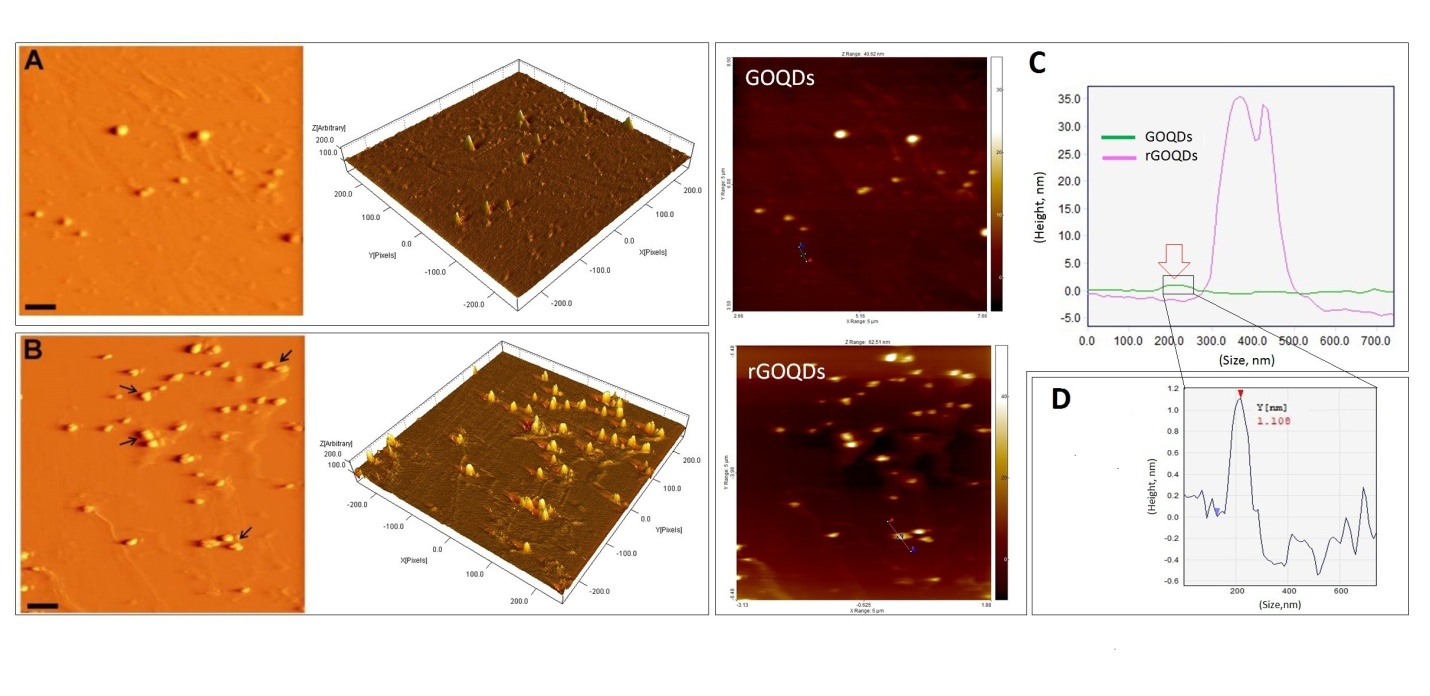


**S1 File Fig D.** Congo red binding absorption spectra of bovine insulin in the absence and presence of various concentrations of GOQDs (A) and rGOQDs (B). Congo red absorbance alone and in the presence of native bovine insulin are also indicated.


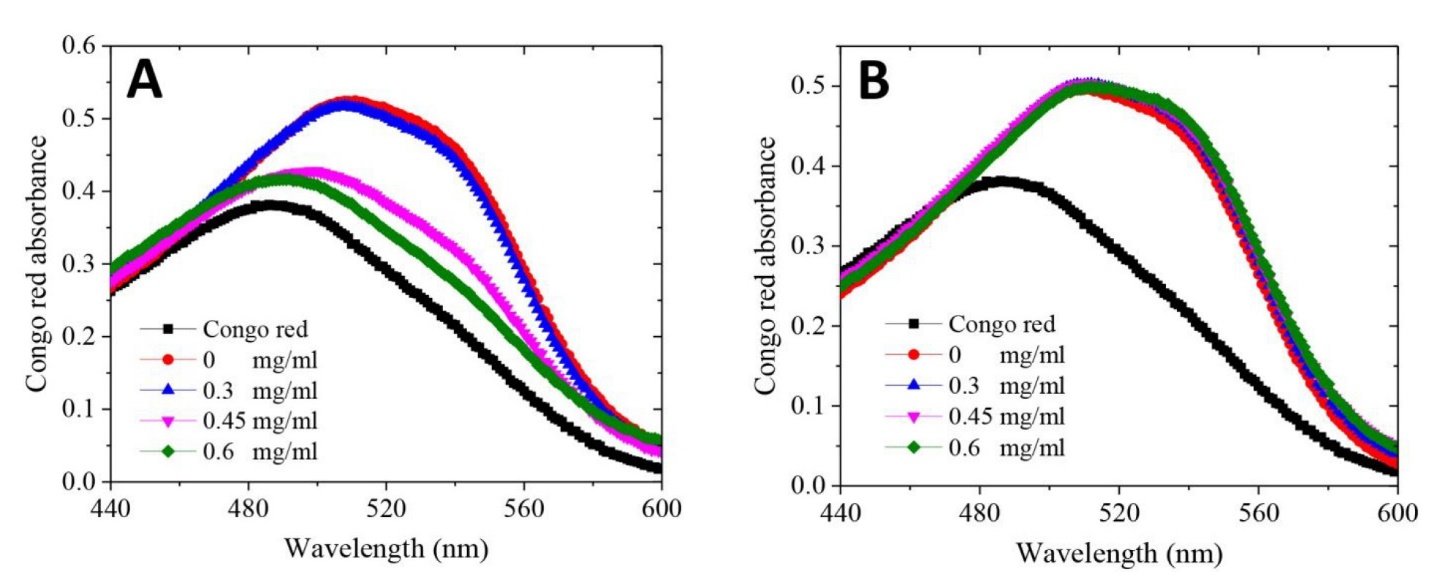


**S1 File Fig E.** Line profiles of AFM images relating to Fig 4H, I, and L.


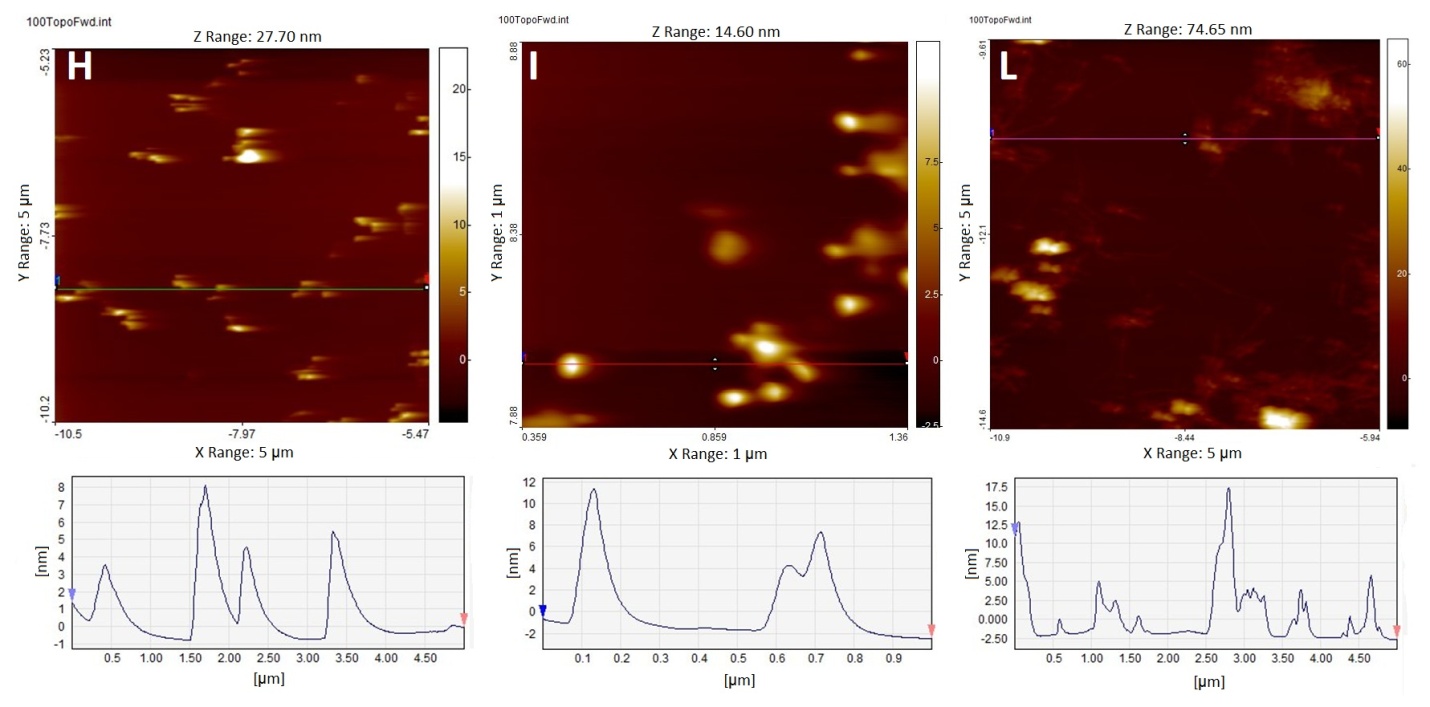


**S1 File Fig F.** Congo red binding absorption spectra of HEWL in the absence and presence of various concentrations of GOQDs (A) and rGOQDs (B). Congo red absorbance alone and in the presence of native HEWL are also indicated.


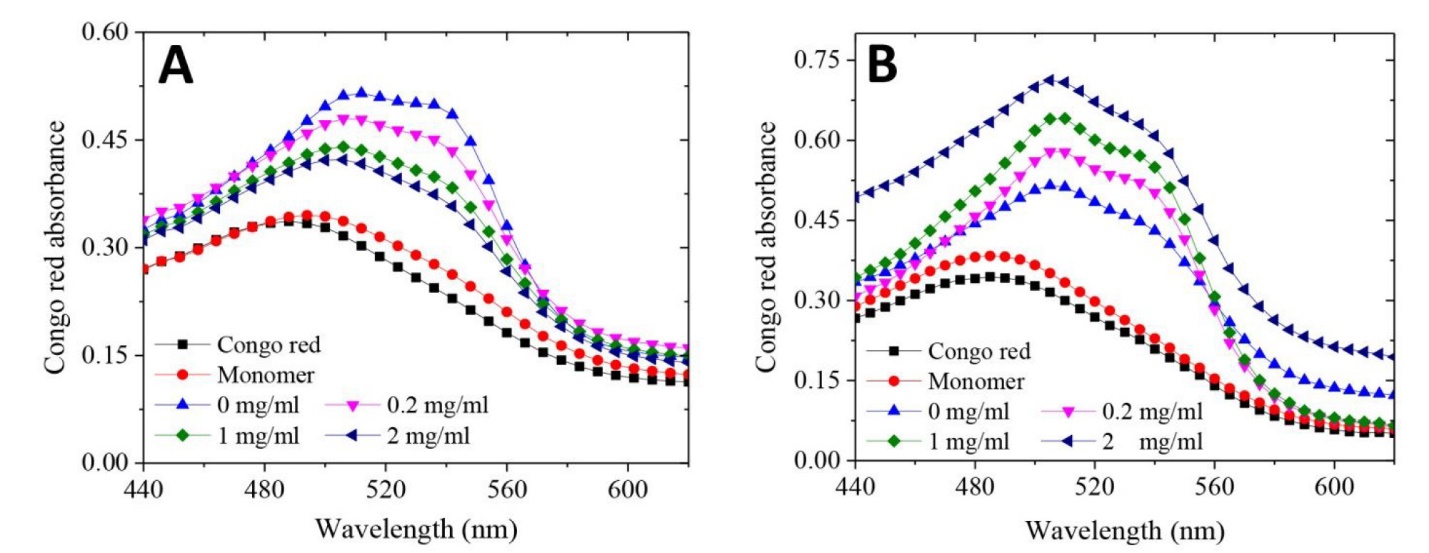


**S1 File Fig G.** Effect of various concentrations of GOQDs and rGOQDs on the viability of SH-SY5Y cells.


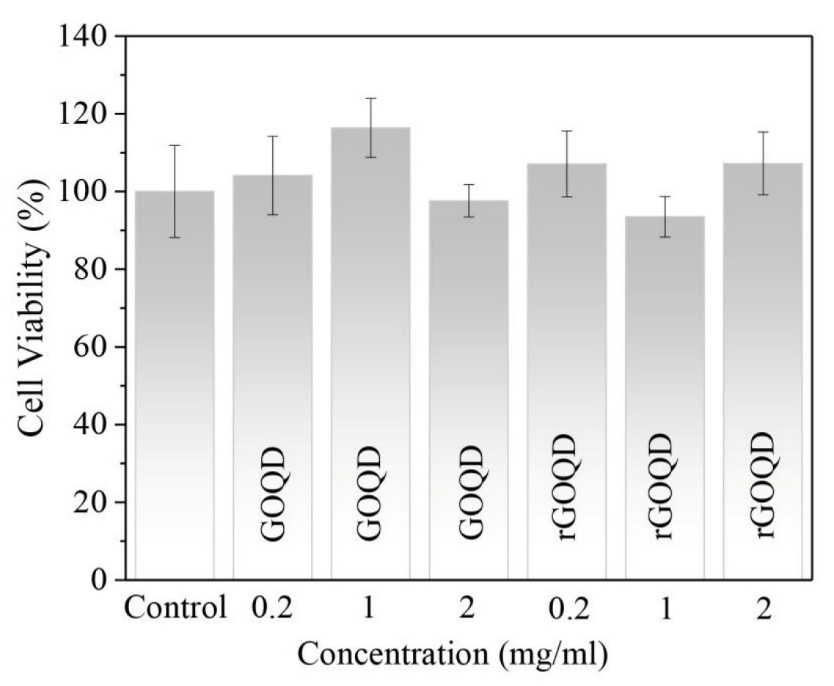

Supplement: S1 File — Fig A. TEM images of rGOQDs acquired from different preparations. Fig B. UV−Vis absorption of 0.3 mg/ml of GOQDs and rGOQDs aqueous solutions. Fig C. AFM and 3D topographical images of GOQDs (A) and rGOQDs (B) and related line profiles (C). (D) An enlarged view of GOQDs line profile. The arrows in (B) indicate some aggregates formed upon reduction. The scale bars represent 500 nm. Fig D. Congo red binding absorption spectra of bovine insulin in the absence and presence of various concentrations of GOQDs (A) and rGOQDs (B). Congo red absorbance alone and in the presence of native bovine insulin are also indicated. Fig E. Line profiles of AFM images relating to Fig 4H, 4I and 4L. Fig F. Congo red binding absorption spectra of HEWL in the absence and presence of various concentrations of GOQDs (A) and rGOQDs (B). Congo red absorbance alone and in the presence of native HEWL are also indicated. Fig G. Effect of various concentrations of GOQDs and rGOQDs on the viability of SH-SY5Y cells. (DOCX) [file pone.0244296.s001.docx]
